# Prevalence of the imposter phenomenon among chiropractors in South Africa: a cross-sectional survey

**DOI:** 10.1186/s12998-026-00665-9

**Published:** 2026-07-03

**Authors:** Tasmiya Hassen, Christopher Yelverton

**Affiliations:** Department of Chiropractic, Faculty of Health Sciences, University of Johannesburg, John Orr Building, 7th Floor, 55 Beit Street, Doornfontein, Johannesburg, 2028 South Africa

**Keywords:** Imposter phenomenon, Chiropractic, Mental health, Cross-sectional survey, South Africa, Clance imposter phenomenon scale

## Abstract

**Background:**

The Imposter Phenomenon (IP)—doubting one’s abilities despite clear achievements—is well-documented among healthcare professionals but has never been studied among practicing chiropractors, who face unique challenges including professional isolation and ambiguous healthcare identity. This study aimed to determine IP prevalence among South African chiropractors and investigate associations with age, gender, years of experience, and province.

**Methods:**

A cross-sectional survey using the validated Clance Imposter Phenomenon Scale (CIPS) was distributed via email to all 960 chiropractors registered with the Allied Health Professions Council of South Africa. Data were collected online from 25 March to 17 May 2024. Descriptive statistics, independent-samples t-tests, and one-way ANOVA were used; results are interpreted through effect sizes (Cohen’s d) and 95% confidence intervals, with exact p-values reported as continuous evidence measures. Cronbach’s alpha assessed reliability. The study followed STROBE and CHERRIES guidelines.

**Results:**

Of 159 chiropractors (16.6% response rate), mean age was 37.8 years (SD = 10.8); 61.6% were female. Mean CIPS score was 50.78 (SD = 16.82), indicating moderate IP. Severity distribution: 33.3% few, 39.0% moderate, 22.6% frequent, 5.0% intense IP (66.6% at or above moderate threshold). Females scored higher than males (55.66 vs. 42.93; t(147.69)= − 5.25, *p* < 0.001; d = 0.85). Chiropractors  ≤ 35 years scored higher than those  > 35 years (53.52 vs. 47.95; t(152) = 2.05, *p* = 0.042; d = 0.33). Chiropractors with ≤ 10 years of experience showed a non-significant trend toward higher IP scores than those with  > 10 years (52.90 vs. 47.94; two-tailed *p* = 0.066; d = 0.30). No provincial differences were found (F(3,155) = 0.62, *p* = 0.601). Cronbach’s α was 0.94.

**Conclusions:**

Moderate to intense IP affects two-thirds of chiropractors in this South African sample, particularly females and early-career practitioners. These findings highlight the need for targeted mental health interventions, mentorship, and peer support. This first study of IP among practicing chiropractors provides evidence for practitioner well-being initiatives with implications for chiropractic education, professional development, and retention.

## Background

The Imposter Phenomenon (IP) describes the experience of doubting one’s abilities despite clear achievements, often accompanied by a persistent fear of being exposed as a fraud [1]. Individuals experiencing IP attribute their success to external factors such as luck or timing rather than their own competence, leading to significant psychological distress. This phenomenon is particularly prevalent among high-achieving individuals in demanding professional environments.

In healthcare professions, IP has been extensively documented. A recent systematic review found that IP affects a substantial proportion of medical students, physicians, and nurses, with reported prevalence rates ranging from 22 to 60% [2]. The high-stakes nature of clinical decision-making, combined with the expectation of expertise and fear of error, creates an environment conducive to imposter feelings. Left unaddressed, IP can contribute to burnout, anxiety, depression, and reduced job satisfaction, ultimately affecting both practitioner well-being and patient care quality.

Chiropractors face unique professional challenges that may increase their susceptibility to IP. Unlike many healthcare professionals who work in large institutional settings, chiropractors often practice in semi-independent or solo practice environments, which can lead to professional isolation and reduced opportunities for peer feedback and mentorship [3, 4]. Additionally, chiropractic occupies a dual position in healthcare, recognized as both a mainstream musculoskeletal profession and a complementary and alternative medicine discipline [5]. This ambiguous professional identity may contribute to self-doubt and feelings of inadequacy, particularly among practitioners who question whether their training and skills are valued by the broader healthcare system. Beyond professional isolation, South African chiropractors face additional stressors, including financial pressures associated with establishing a private practice, job insecurity inherent to entrepreneurial healthcare models, and the absence of a public sector safety net. These factors may compound feelings of self-doubt and inadequacy, potentially increasing susceptibility to IP.

While IP has been studied among chiropractic students [4, 6], no studies to date have examined IP among practicing chiropractors. Furthermore, the chiropractic profession in South Africa remains under-researched regarding practitioners’ mental health and well-being. Given the unique professional landscape of chiropractic in South Africa, including regulatory oversight by the Allied Health Professions Council of South Africa (AHPCSA) and a diverse patient population [3], understanding IP prevalence in this context is important for developing targeted support interventions.

Based on the existing literature, this study tested the following hypotheses: (1) female chiropractors would report higher IP scores than male chiropractors, consistent with research showing higher IP prevalence among women in healthcare [1, 7]; (2) chiropractors aged 35 years or younger would report higher IP scores than those older than 35 years; (3) chiropractors with fewer than 10 years of experience would report higher IP scores than those with more than 10 years of experience, as early-career practitioners may experience greater professional uncertainty [7]; and (4) no significant differences in IP scores would be observed across provinces of practice, serving as a null hypothesis to confirm that IP is not geographically concentrated.

Accordingly, the primary aim of this study was to determine the prevalence of the Imposter Phenomenon among chiropractors in South Africa and investigate how demographic factors, including age, gender, and years of experience, influence IP experiences.

## Methods

### Study design

This study used a cross-sectional survey design to assess the prevalence of the Imposter Phenomenon (IP) among chiropractors in South Africa.

### Participants

The study population included all 960 chiropractors registered with the Allied Health Professions Council of South Africa (AHPCSA, 2024). Emails with a survey link were distributed to all registered chiropractors via the AHPCSA and the Chiropractic Association of South Africa (CASA) mailing lists.

A target sample size of 100 participants was established a priori based on recommendations for cross-sectional survey research, which indicate that correlation analyses and t-tests require a minimum of 100–150 observations to produce stable parameter estimates and adequate statistical power [8]. Based on the total population of 960 registered chiropractors, a target of 100 participants represented approximately 10% of the population. The achieved sample of 159 participants exceeded this target.

### Inclusion criteria

Inclusion criteria were: (1) currently registered with the AHPCSA; and (2) actively practicing in South Africa at the time of the study. No exclusion criteria were applied.

### Survey instrument

The survey was divided into two sections:

Section A collected demographic information, including age, gender, years in practice, and province of practice. Demographic variables (age, gender, years in practice, province) were selected based on prior IP literature identifying these factors as potential predictors [2]. Self-reported gender was collected, acknowledging the distinction between sex and gender.

Section B comprised the Clance Imposter Phenomenon Scale [1, 9], a validated 20-item instrument that assesses the intensity of IP experiences. The original, unmodified CIPS was used [10]. Participants rated their agreement with each statement on a 5-point Likert scale (1 = “Not at all true” to 5 = “Very true”). Total scores range from 20 to 100, with higher scores indicating more frequent IP experiences. Standard CIPS cutoffs [9] were used to categorize IP severity: <41 = few IP characteristics, 41–60 = moderate IP experiences, 61–80 = frequent IP feelings, and > 80 = intense IP experiences.

### Data collection

The survey was administered online via QuestionPro from 25 March 2024 to 17 May 2024. Mean survey completion time was 7 min. Participants were required to provide informed consent before completing the survey. Responses were anonymous, and only fully completed surveys were included in the analysis. A single reminder email was sent two weeks after the initial invitation.

### Statistical analysis

Data were analysed using SPSS (version 29). Descriptive statistics (frequencies, percentages, means, and standard deviations) were used to summarise demographic information and IP scores.

To assess differences in IP scores between groups, independent-samples t-tests were used for binary categorical variables (gender, age dichotomised at 35 years, years of practice dichotomised at 10 years). The cut-point of 35 years was pre-specified based on prior IP literature [2, 11] and coincided with the sample median. A sensitivity analysis treating age as continuous was also conducted. Levene’s test for equality of variances was performed, and equal variances were not assumed when indicated. To compare IP scores by years of practice, a one-tailed test was used, based on the a priori directional hypothesis that chiropractors with fewer years of experience would report higher IP scores. All other comparisons used two-tailed tests. Effect sizes were calculated using Cohen’s d.

A one-way ANOVA was used to examine differences in IP scores across provinces of practice, with small provinces combined into an “Other” category.

Cronbach’s alpha was used to assess the internal consistency reliability of the CIPS. Results are interpreted based on the magnitude of effect sizes (Cohen’s d) and the precision of estimates (95% CIs), with exact *p*-values reported as continuous measures of data-model compatibility rather than as binary significance indicators.

### Ethical considerations

The study was approved by the Faculty of Health Sciences Research Ethics Committee at the University of Johannesburg (REC-2480-2023). All participants provided informed consent by clicking a button to proceed to the survey after reading the information letter. Participants were informed that their responses would be anonymous, that no identifying information would be collected, and that they could withdraw at any time by closing their browser. Data were stored on a password-protected university server accessible only to the research team.

### Reporting guidelines

This study followed the STROBE guidelines for cross-sectional studies and the CHERRIES guidelines for web-based surveys. Specific CHERRIES items addressed include: (1) informed consent obtained via click-to-proceed; (2) duplicate responses prevented via IP address and cookie tracking; (3) only fully completed surveys included in analysis; and (4) survey duration of 54 days with one reminder email.

## Results

### Response rate and demographic characteristics

A total of 159 chiropractors participated in the survey, yielding a minimum response rate of 16.6% (159/960 registered chiropractors). All 159 responses were fully completed for the CIPS items, however, age data were missing for 5 participants.

Demographic characteristics of the sample are presented in Table 1. Of the respondents, 61.6% (*n* = 98) were female, and 38.4% (*n* = 61) were male. Participant ages ranged from 25 to 84 years, with a mean age of 37.8 years (SD = 10.8; median = 35.5). For subgroup analysis, age was dichotomised at 35 years, resulting in 50.0% (*n* = 77) aged 35 years or younger and 50.0% (*n* = 77) aged 35 years or older (5 participants missing).

In terms of professional experience, 34.0% (*n* = 54) had been practicing for less than 5 years, 23.3% (*n* = 37) for 5–10 years, 11.3% (*n* = 18) for 11–15 years, 9.4% (*n* = 15) for 16–20 years, and 22.0% (*n* = 35) for more than 20 years. For subgroup analysis, years of practice were dichotomised at 10 years: 57.2% (*n* = 91) had 10 years or less of experience, and 42.8% (*n* = 68) had more than 10 years.

Regarding geographic distribution, the majority of respondents practiced in Gauteng (47.8%, *n* = 76), followed by KwaZulu-Natal (24.5%, *n* = 39) and the Western Cape (17.6%, *n* = 28). Smaller proportions practiced in the Eastern Cape, Limpopo, and Mpumalanga (2.5% each), Free State (1.3%), and Northern Cape and North West (0.6% each).

**Table 1:** Demographic characteristics of participants (*N* = 159)

Characteristic	Category	*n*	%
Gender	Male	61	38.4
	Female	98	61.6
Age	Mean (SD)	37.8 (10.8) years	
	Range	25–84 years	
	≤ 35 years	77	50.0*
	> 35 years	77	50.0*
Years in practice	< 5 years	54	34.0
	5–10 years	37	23.3
	11–15 years	18	11.3
	16–20 years	15	9.4
	> 20 years	35	22.0
Years in practice (dichotomised)	≤ 10 years	91	57.2
	> 10 years	68	42.8
Province of practice	Gauteng	76	47.8
	KwaZulu-Natal	39	24.5
	Western Cape	28	17.6
	Eastern Cape	4	2.5
	Limpopo	4	2.5
	Mpumalanga	4	2.5
	Free State	2	1.3
	Northern Cape	1	0.6
	North West	1	0.6

*Age data missing for 5 participants; percentages calculated based on *n* = 154 for age variables

### Imposter phenomenon scores

**Fig. 1:**
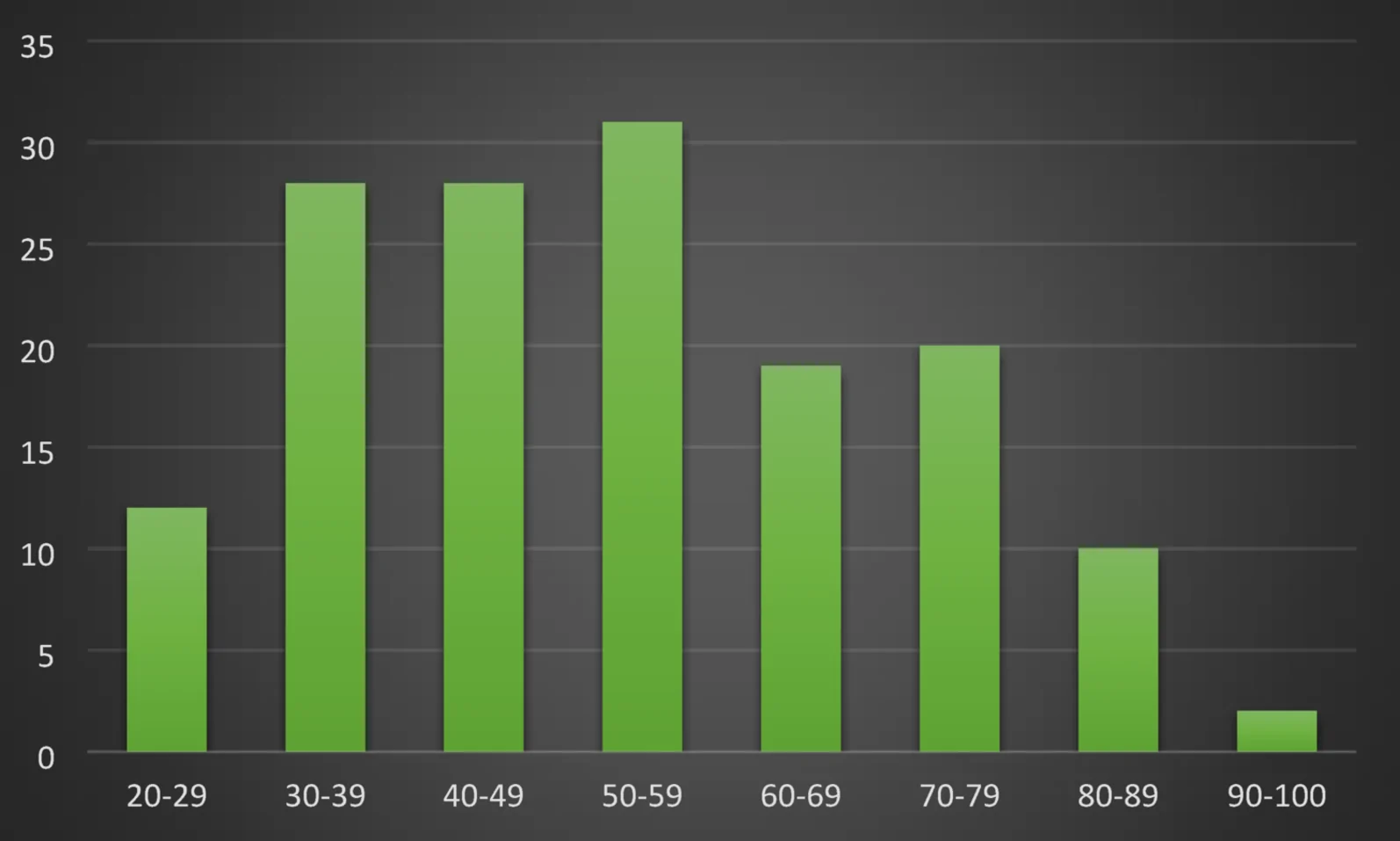
Distribution of Clance Imposter Phenomenon Scale (CIPS) total scores among South African chiropractors (*N* = 159). Higher scores indicate more frequent experiences of the Impostor Phenomenon

The mean Imposter Phenomenon (IP) score on the Clance Imposter Phenomenon Scale (CIPS) was 50.78 (SD = 16.82, range: 20–95, median = 48.00), indicating moderate IP experiences across the sample. The distribution of IP scores is presented in Fig. 1.

Using standard CIPS cutoffs [9], the severity of IP experiences was categorized as follows:

- Few IP characteristics (score < 41): 33.3% (*n* = 53).
- Moderate IP experiences (score 41–60): 39.0% (*n* = 62).
- Frequent IP feelings (score 61–80): 22.6% (*n* = 36).
- Intense IP experiences (score > 80): 5.0% (*n* = 8).

**Fig. 2:**
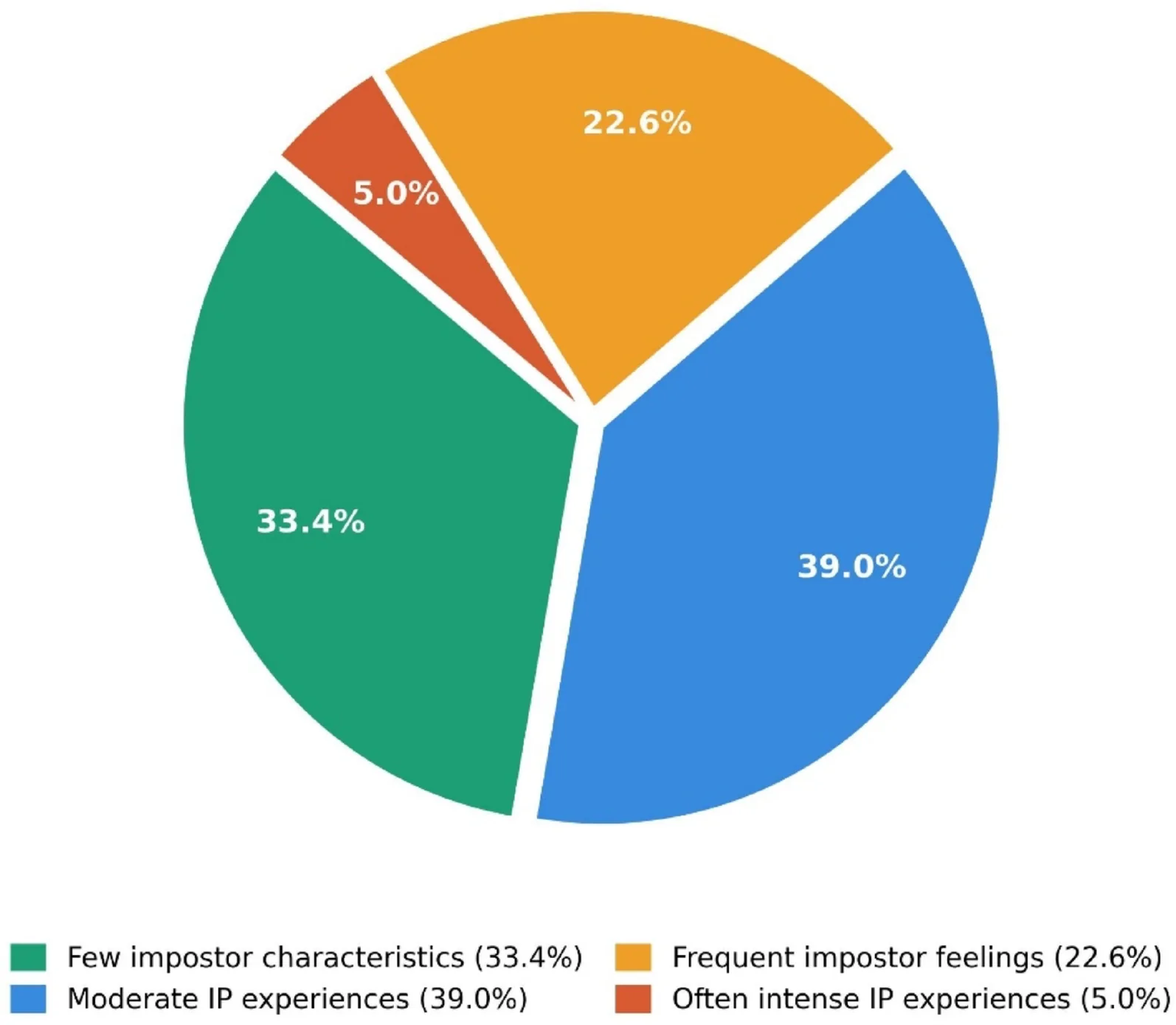
Distribution of imposter phenomenon severity categories among South African chiropractors (*N* = 159)

Thus, 66.6% (n = 106) of participants scored at or above the moderate IP threshold (Table 2 and Fig. 2).

**Table 2:** Distribution of imposter phenomenon severity

Severity level	Score range	*n*	%
Few IP characteristics	< 41	53	33.3
Moderate IP experiences	41–60	62	39.0
Frequent IP feelings	61–80	36	22.6
Intense IP experiences	> 80	8	5.0
Total		159	100.0

Using the traditional CIPS cut-off score of 62 to indicate the presence of Imposter Phenomenon [10], 26.4% (*n* = 42) of participants scored at or above this threshold.

### Scale reliability

The Clance Imposter Phenomenon Scale demonstrated excellent internal consistency in this sample. Cronbach’s alpha was 0.94 for the 20-item scale, well above the commonly accepted threshold of 0.70 [12]. Corrected item-total correlations ranged from 0.386 to 0.843, indicating that all items contributed positively to the scale’s reliability [13].

### Gender differences in IP scores

Female chiropractors reported substantially higher IP scores (M = 55.66, SD = 16.92) than male chiropractors (M = 42.93, SD = 13.46), a mean difference of 12.73 points (95% CI: 7.93 to 17.53). Levene’s test indicated unequal variances between groups (F = 6.85, *p* = 0.010), so equal variances were not assumed. The t-test result was t(147.69) = − 5.25, *p* < 0.001, with a mean difference of − 12.73 (95% CI: − 17.53 to − 7.93). Cohen’s d was 0.85, representing a large effect size (Table 3).

**Table 3:** IP scores by gender

Gender	*n*	Mean IP score (SD)	95% CI	t	df	*p*	Cohen’s d
Male	61	42.93 (13.46)	39.49–46.38	− 5.25	147.69	< 0.001	0.85
Female	98	55.66 (16.92)	52.27–59.06				

### Age differences in IP scores

As presented in Table 4, Chiropractors aged 35 years or younger reported higher IP scores (M = 53.52, SD = 15.89) than those older than 35 years (M = 47.95, SD = 17.84), a men difference of 5.57 points (95% CI: 0.19 to 10.95; t(152) = 2.05, *p* = 0.042). Levene’s test indicated equal variance (F = 0.89, *p* = 0.348). A sensitivity analysis treating age as a continuous variable confirmed the inverse relationship between age and IP scores (Pearson’s *r* = − 0.18, *p* = 0.026). Cohen’s d was 0.33, representing a small to moderate effect size.

**Table 4:** IP scores by age group

Age Group	*n*	Mean IP score (SD)	95% CI	t	df	*p*	Cohen’s d
≤ 35 years	77	53.52 (15.89)	49.91–57.13	2.05	152	0.042	0.33
> 35 years	77	47.95 (17.84)	43.90–52.00				

Age data missing for 5 participants (*n* = 154 for this analysis)

### Years of practice differences in IP scores

Chiropractors with 10 years or less of experience reported higher IP scores (M = 52.90, SD = 16.06) than those with more than 10 years of experience (M = 47.94, SD = 17.52). An independent-samples t-test assuming equal variances (Levene’s F = 0.26, *p* = 0.609) showed a non-significant trend in the hypothesized direction: t(157) = 1.85, two-tailed *p* = 0.066. The mean difference was 4.96 (95% CI: − 0.33 to 10.25). The confidence interval crossed zero, consistent with the non-significant result. Cohen’s d was 0.30, representing a small effect size with priori directional hypothesis. (Table 5).

**Table 5:** IP scores by years of practice

Experience group	*n*	Mean IP score (SD)	95% CI	t	df	*p* (two-tailed)	Cohen’s d
≤ 10 years	91	52.90 (16.06)	49.56–56.24	1.85	157	0.066	0.30
> 10 years	68	47.94 (17.52)	43.70–52.18				

### Provincial differences in IP scores

A one-way ANOVA was conducted to examine whether IP scores differed significantly across provinces of practice. Due to small sample sizes in several provinces (Northern Cape, North West, Free State, Eastern Cape, Limpopo, Mpumalanga), these were combined into an “Other” category for analysis. The analysis included four groups: Gauteng (*n* = 76), KwaZulu-Natal (*n* = 39), Western Cape (*n* = 28), and Other (*n* = 16) (Table 6). No significant differences in IP scores were observed across provinces, F(3, 155) = 0.62, *p* = 0.601.

**Table 6:** IP scores by province of practice

Province	*n*	Mean IP score (SD)
Gauteng	76	51.21 (17.28)
KwaZulu-Natal	39	51.08 (16.33)
Western Cape	28	49.32 (15.92)
Other (combined)	16	48.44 (17.45)

F(3, 155) = 0.62, *p* = 0.601

### Summary of key findings

The main findings of this study are summarized in Table 7 below. In brief:

1. The mean IP score was 50.78 (SD = 16.82), with 66.6% of participants scoring at or above the moderate IP threshold.
2. Female chiropractors reported significantly higher IP scores than males (mean differences 12.73 points; large effect size, d = 0.85).
3. Chiropractors aged 35 years or younger reported significantly higher IP scores than older chiropractors (small-moderate effect size, d = 0.33).
4. Chiropractors with 10 years or less of experience reported a non-significant trend toward higher IP scores than those with more experience (small effect size, d = 0.30; two-tailed *p* = 0.066).
5. No significant differences in IP scores were observed across provinces of practice.

**Table 7:** Summary of IP scores by demographic subgroup

Variable	Category	*n*	Mean IP score (SD)	Comparison	Test statistic	*p*-value	Effect size
Gender	Male	61	42.93 (13.46)	Female vs. Male	t(147.69) = − 5.25	< 0.001	d = 0.85
	Female	98	55.66 (16.92)				
Age	≤ 35 years	77	53.52 (15.89)	Younger vs. Older	t(152) = 2.05	0.042	d = 0.33
	> 35 years	77	47.95 (17.84)				
Years of practice	≤ 10 years	91	52.90 (16.06)	Less vs. More	t(157) = 1.85	0.033*	d = 0.30
	> 10 years	68	47.94 (17.52)				
Province	Gauteng	76	51.21 (17.28)	Across provinces	F(3,155) = 0.62	0.601	η^2^ = 0.01
	KZN	39	51.08 (16.33)				
	Western Cape	28	49.32 (15.92)				
	Other	16	48.44 (17.45)				

Two-tailed *p*-value reported throughout; the year of practice comparison reflects the priori directional hypothesis (d = 0.30)

## Discussion

This study is the first to investigate the Imposter Phenomenon (IP) among chiropractors in South Africa. The findings demonstrate that IP is prevalent among South African chiropractors, with two-thirds of practitioners experiencing moderate to intense imposter feelings. Female chiropractors are at substantially higher risk than their male counterparts, and early-career practitioners (those aged 35 years or younger and those with 10 years or less of experience) report significantly more imposter experiences. These patterns align with international research while highlighting unique contextual factors related to chiropractic’s professional identity in South Africa.

### Prevalence of imposter phenomenon

The majority of chiropractors in this study (66.6%) scored at or above the moderate IP threshold, with 39.0% experiencing moderate IP, 22.6% frequent IP, and 5.0% intense IP. The mean score of 50.78 is comparable to studies in other healthcare professions. Previous studies on physical therapists reported a mean IP score of 57.33 [14], and a mean of 60.3 among 514 physiotherapists [15].

The finding that one-third of chiropractors (33.3%) reported few IP characteristics is notable. One possible explanation is the unique professional identity of chiropractors in South Africa. Unlike physiotherapy, which is well-established within the public healthcare system [16, 17], chiropractors in South Africa are limited to private practice and are not integrated with the public healthcare system [3]. This role ambiguity could, paradoxically, reduce certain pressures. Chiropractors may face less direct comparison to other healthcare providers and fewer opportunities for hierarchical evaluation, potentially mitigating the most intense forms of IP [18, 19].

However, the finding that 66.6% of participants experienced moderate to intense IP indicates that professional uncertainty remains a significant challenge. The evolving status of chiropractic care, perceived by some medical doctors as complementary and alternative medicine (CAM) rather than mainstream medicine [20], can create uncertainty about professional role and value. This uncertainty may contribute to self-doubt, as observed in other professions with less clearly defined roles [1, 21]. The dual influence of professional identity on IP, both mitigating intense feelings while contributing to moderate self-doubt, reflects the complex relationship between role ambiguity and psychological well-being [1].

### Age differences in imposter phenomenon

Chiropractors aged 35 years or younger reported higher IP scores than those older than 35 (53.52 vs. 47.95; *p* = 0.042; d = 0.33). This finding aligns with existing literature. A recent systematic review of IP studies in healthcare found that two studies reported that increased age was associated with decreased impostor feelings [2]. Additional studies have demonstrated a reduction in IP feelings with age and experience [14], and that scores decreased with age, with particularly low scores in the 55–65 year age group [11]. A study targeting young neurosurgeons between the ages of 20 and 40 years found that 94% of respondents exhibited signs of IP, with the majority experiencing moderate (36.21%) or frequent (40.52%) feelings [22].

Age and years of practice were correlated in this sample (Spearman’s ρ ≈ 0.85), consistent with expectations. Therefore, these variables cannot be treated as fully independent predictors of IP. Younger chiropractors are likely in the transition from academic training to independent clinical practice, navigating patient care, business management, and continuing education simultaneously. This transition can be overwhelming and contribute to feelings of being unprepared, a core contributor to IP [9, 23].

The demographic distribution of the South African chiropractic profession may also influence these findings, with the majority of chiropractors under the age of 50 years due to the historic regulation development [3] which explains the younger mean age in this sample and the higher representation of early-career practitioners [24–26].

#### Gender differences in imposter phenomenon

Female chiropractors reported significantly higher IP scores than males (mean differences 12.73 points, 95% CI: 7.93 to 17.53; *p* < 0.001; d = 0.85). The effect size is large, indicating that gender is a substantial predictor of IP in this sample.

This finding is consistent with international research. Female residents scored higher on IP measures than males (61.8 vs. 57.7) [27], and medical students in Pakistan showed 53.5% of those having this feeling were female [28]. Previous studies have documented higher IP prevalence among women [4, 29]. Additionally, 66% of their physiotherapy sample was female, consistent with the higher representation of women in healthcare professions and their greater willingness to participate in psychological research [26, 30, 31]. Importantly, the gender distribution of our sample (61.6% female) is representative of the South African chiropractic profession, which has approximately 59% female practitioners [3, 32]. South Africa is one of the few countries with more female practitioners [3, 32], which naturally increases the likelihood of receiving responses from females.

Several explanations for gender differences in IP have been proposed. Societal expectations and gender roles often place additional pressure on women to perform exceptionally, leading to heightened self-doubt [4]. This gender difference may be partly explained by lower self-esteem and higher neuroticism in women [33]. Women also face challenges in balancing professional responsibilities with family and caregiving duties, which can exacerbate feelings of inadequacy [29]. The underrepresentation of women in leadership positions within healthcare can lead to isolation and self-doubt, as women may struggle to see themselves as deserving of success in environments where they are not equally represented [34]. Additionally, women are more likely to internalize failure, attributing setbacks to personal shortcomings rather than external factors [9, 27].

### Experience differences in imposter phenomenon

Chiropractors with 10 years or less of experience showed a non-significant trend toward higher IP scores than those with more than 10 years (two-tailed *p* = 0.066; d = 0.30). The direction of magnitude (d = 0.30, a small effect) are consistent with the hypothesized pattern and align with the broader literature. A longer duration in a professional role is linked to a decreased likelihood of experiencing IP, with 11–20 years of experience potentially representing an optimal range [15]. Individuals in the early stages of their careers, particularly those with less experience, were more likely to experience IP, feeling that they had not yet “earned” their positions [9]. Medical students and early-career physicians with fewer years of experience exhibited higher levels of imposter feelings [35] and younger age and fewer years of professional experience are consistently associated with higher levels of IP [2]. Studies of librarians, registered dietitians, veterinarians, and academics across multiple fields consistently show that those with less experience report higher levels of IP [36–38].

Early-career professionals face a steep learning curve as they transition from theoretical knowledge to real-world practice. The lack of extensive clinical experience can exacerbate self-doubt, particularly when comparing themselves to more experienced colleagues [39]. The pressures and expectations placed on healthcare professionals, including chiropractors, can contribute to IP, with young chiropractors feeling an intense need to prove themselves in a competitive field. This pressure is often compounded by the lack of a strong support system, as younger practitioners may not yet have established networks of mentors or peers to provide guidance and reassurance [40, 41].

#### Comparison with global trends

The IP prevalence observed among South African chiropractors is broadly consistent with international studies of healthcare professionals. Previous studies [2, 11, 15] have documented IP across diverse healthcare settings, with early-career practitioners and women consistently reporting higher levels. This consistency suggests that common stressors such as high expectations, professional transitions, and societal norms regarding success and achievement, transcend national boundaries [41].

However, contextual factors specific to South Africa may influence how IP manifests. Cultural attitudes towards healthcare providers, the unique regulatory history of chiropractic in South Africa, and the profession’s ongoing negotiation of its identity within the healthcare system may shape both the experience and expression of imposter feelings. Understanding these nuances can inform culturally sensitive interventions to help chiropractors navigate and mitigate IP. However, current evidence for IP interventions remains inconclusive, with a recent scoping review identifying limited high-quality studies in this area [42].

### Limitations

Several limitations should be considered when interpreting these findings. The 16.6% response rate may contribute to non-response bias [25, 30] and chiropractors experiencing higher or lower IP may have been differentially likely to respond. Current concepts related to survey response rates accept that lower response rates (15%), are in many cases, still valid [43], and specifically if the results, as is the case in this research, are still representative of the target population [44]. Furthermore, this response rate is comparable to recent web-based surveys of chiropractors published in peer-reviewed journals; for example, a survey of Canadian sport and exercise physicians on attitudes towards chiropractors reported an 11% response rate, while a survey of UK chiropractors achieved 17.1% [45, 46]. The wide range of response rates in chiropractic survey research (9–76%) reflects the challenges of engaging busy practitioners in voluntary, web-based surveys without mandatory compliance. The demographic characteristics of our sample (57% with ≤ 10 years of experience; 62% female) align with known population parameters of the South African chiropractic profession [3, 32], supporting the representativeness of our findings.

The cross-sectional design precludes causal inferences, and we cannot determine whether experience reduces IP over time or whether individuals with lower IP simply remain in practice longer. Self-reported data are subject to social desirability bias, and participants may have underreported imposter feelings due to stigma. The sample was drawn from a single country, limiting generalizability to chiropractors in other regulatory environments. The dichotomization of age at 35 years and experience at 10 years, while providing clinically interpretable groups, may have obscured non-linear relationships. The study did not collect data on practice setting (solo vs. group practice) or patient volume, which may influence IP experiences.

### Implications for practice and research

These findings suggest that targeted interventions for IP may be warranted, particularly for female, younger, and less experienced chiropractors. Chiropractic training programs and professional associations should consider incorporating IP awareness into continuing education and mentorship programs, establishing peer support groups where practitioners can discuss imposter feelings without stigma, providing structured mentorship for early-career chiropractors, pairing them with experienced practitioners, and normalising discussions of professional self-doubt in clinical training environments.

Future research should employ longitudinal designs to track IP throughout chiropractors’ careers and determine whether IP decreases with experience or whether individuals with high IP exit the profession. Qualitative research could explore the specific sources of IP in chiropractic practice, including the role of professional identity ambiguity, practice isolation, and referral relationships with medical practitioners. Cross-national comparative studies would help determine whether IP prevalence varies across regulatory environments and levels of professional integration into public healthcare systems. Finally, intervention studies should evaluate whether mentorship programs, peer support groups, or cognitive reframing techniques reduce IP and improve practitioner well-being.

## Conclusion

This study demonstrates that moderate to intense Imposter Phenomenon experiences are prevalent among two-thirds of chiropractors in this South African sample, with female, younger, and less experienced practitioners at the highest risk. These findings align with international IP research in healthcare professions while highlighting unique contextual factors related to chiropractic’s professional identity in South Africa. The large gender effect (d = 0.85) is particularly noteworthy and warrants targeted intervention. Addressing IP through mentorship, peer support, and curriculum changes may improve professional confidence and practitioner well-being, with potential downstream benefits for patient care.

## Data Availability

No datasets were generated or analysed during the current study.
